# Supplementary material for: Content-rich biological network constructed by mining PubMed abstracts
Source: BMC Bioinformatics. 2004 Oct 8;5:147. doi: 10.1186/1471-2105-5-147 (PMC528731; doi:10.1186/1471-2105-5-147)
Supplement: Additional File 2 — The original results of the above study (non-essential files are deleted to keep the file size under the limit set by BMC bioinformatics). [file 1471-2105-5-147-S2.bz2 › chilibotAdditionalFile2/dip30/217ID7744001E725/html/IL6ST_IL6.html]

 


 **IL6ST** and **IL6** 
  
Found 848 abstracts in PubMed, retrieved 30.  
 

 What does Google say? 
 PDF only 
| .edu only 

---

**Interactive relationship** (e.g. stimulation, inhibition, etc)

**Neutral relationship**- AIMS Interleukin 6  [ **IL6** ]  IL 6  [ **IL6** ]  contributes via its signal transducer gp130  [ **IL6ST** ]  to the acute phase response APR in hepatocytes.  Ref: 12891556 Gastroenterology, 2003
- Ciliary neurotrophic factor CNTF is a gp130  [ **IL6ST** ]  neuroregulatory cytokine belonging to the interleukin 6  [ **IL6** ]  type cytokine superfamily.  Ref: 12890930 Gynecol Obstet InvestGynecol Obstet Invest, 2003
- Thus, constitutive activation of STAT3 in HNSCC may use an autocrine paracrine activating loop mediated by  **IL6**  and other cytokines acting through the gp130  [ **IL6ST** ]  receptor family, which may confer both proliferative and survival potential in this malignancy.  Ref: 12782602 Cancer Res, 2003
- IL 11 belongs to the gp130  [ **IL6ST** ]  cytokine family, which includes also IL 6  [ **IL6** ] .  Ref: 12697042 J Endocrinol, 2003
- The IL 6  [ **IL6** ]  receptor is composed of two different subunits, an alpha subunit IL 6Ralpha that binds IL 6  [ **IL6** ]  with low affinity and a beta subunit gp130  [ **IL6ST** ]  that binds the IL 6  [ **IL6** ] .IL 6Ralpha complex with high affinity and as a result triggers intracellular signaling.  Ref: 12646580 J Biol Chem, 2003
- In its soluble form, gp130  [ **IL6ST** ]  is a natural antagonist that neutralizes IL 6  [ **IL6** ] .soluble IL 6Ralpha complexes.  Ref: 12646580 J Biol Chem, 2003
- One good illustration is derived from the in vitro observations that IL 6  [ **IL6** ]  promotes the growth arrest and differentiation of M1 cells through gp130  [ **IL6ST** ]  mediated STAT3 activation.  Ref: 12687404 Rev Physiol Biochem Pharmacol< MedlineTA>Rev Physiol Biochem Pharmacol, 2003
- Previous studies showed that activation of the gp130  [ **IL6ST** ]  receptor system, using as ligand interleukin 6  [ **IL6** ]  fused to its soluble receptor IL6RIL6, causes induction of myelin genes such as myelin basic protein MBP and myelin protein zero Po in embryonic dorsal root ganglia Schwann cells.  Ref: 12643284 J Biol Chem, 2003
- In conclusion, our experiments indicate an involvement of IL 6  [ **IL6** ]  in the pathogenesis of liver diseases and suggest a protective role of IL 6  [ **IL6** ]  gp130  [ **IL6ST** ]  dependent pathways in nonparenchymal liver cells during fibrosis progression in chronic liver diseases.  Ref: 12830005 Hepatology< MedlineTA>Hepatology, 2003
- Interleukin 6  [ **IL6** ]  IL 6  [ **IL6** ]  is an immunoregulatory cytokine that activates a cell surface signaling assembly composed of IL 6  [ **IL6** ] , the IL 6  [ **IL6** ]  alpha receptor IL 6Ralpha, and the shared signaling receptor gp130  [ **IL6ST** ] .  Ref: 12829785 Science, 2003
- In a target cell, IL 6  [ **IL6** ]  can simultaneously generate functionally distinct or sometimes contradictory signals through its receptor complex, IL 6Ralpha and gp130  [ **IL6ST** ] .  Ref: 12687404 Rev Physiol Biochem Pharmacol< MedlineTA>Rev Physiol Biochem Pharmacol, 2003
- The effects of IL 6  [ **IL6** ]  are mediated through a specific receptor complex made up of a ligand binding glycoprotein gp80 or IL 6R and a signal transducing glycoprotein gp130  [ **IL6ST** ] .  Ref: 12664314 Eur Arch Psychiatry Clin Neurosci, 2003
- The subsequent recruitment of the common signal transducer gp130  [ **IL6ST** ]  and activation of the JAK STAT and SHP 2 Ras mitogen activated protein kinase MAPK pathways are responsible for the majority of cellular responses elicited by IL 6  [ **IL6** ]  cytokines.  Ref: 12764151 J Biol Chem< MedlineTA>J Biol Chem, 2003
- IL 6  [ **IL6** ]  type cytokines exert their action via the signal transducers gp130  [ **IL6ST** ] , LIF receptor and OSM receptor leading to the activation of the JAK STAT and MAPK cascades.This review focuses on recent progress in the understanding of the molecular mechanisms of IL 6  [ **IL6** ]  type cytokine signal transduction.  Ref: 12773095 Biochem J< MedlineTA>Biochem J, 2003
- In this respect murine fibroblasts lacking exon 3 of SHP2 are not sensitive to TNF alpha, indicating that functional SHP2 and its recruitment to gp130  [ **IL6ST** ]  are key events in inhibition of IL 6  [ **IL6** ]  dependent STAT activation by TNF alpha.  Ref: 12817006 J Immunol, 2003
- We previously demonstrated that interleukin 6  [ **IL6** ]  related cytokine, leukemia inhibitory factor LIF, induced cardiac hypertrophy through gp130  [ **IL6ST** ] .  Ref: 12855672 Circ Res< MedlineTA>Circ Res, 2003
- gp130  [ **IL6ST** ]  is a signal transducing receptor component shared by all the IL 6  [ **IL6** ]  family cytokines, whose gene ablation in mouse results in the significant reduction in the fetal liver hematopoiesis.  Ref: 12691915 Exp Hematol, 2003
- The mRNAs of both receptors of IL 6  [ **IL6** ] , the soluble IL6R and gp130  [ **IL6ST** ] , were increased subsequently to IL 6  [ **IL6** ]  mRNA.  Ref: 12733076 Pflugers Arch< MedlineTA>Pflugers Arch, 2003

**Non-interactive relationship** (e.g. studied together, co-existance, homology, etc.)

- A Fusion Protein of the gp130  [ **IL6ST** ]  and Interleukin 6Ralpha Ligand binding Domains Acts as a Potent Interleukin 6  [ **IL6** ]  Inhibitor.  Ref: 12646580 J Biol Chem, 2003
- In the non lineage specific gp130  [ **IL6ST** ]  cytokine family, IL 6  [ **IL6** ]  appears to predominate for the induction of megakaryopoiesis.  Ref: 12634936 Rheumatol Int, 2003
- Here we show that phosphorylation of STAT3 is prolonged in mouse Socs3 deficient macrophages after stimulation with interleukin 6  [ **IL6** ]  IL 6  [ **IL6** ]  but not IL 10, indicating that SOCS3 specifically affects signaling mediated by IL 6  [ **IL6** ]  and gp130  [ **IL6ST** ] .  Ref: 12754506 Nat Immunol< MedlineTA>Nat Immunol, 2003
- However, in situations where a signaling competent receptor is bound at IL 6  [ **IL6** ]  site I, ligand binding to site III is an absolute requirement for participation of the receptor in a signaling heterodimer with gp130  [ **IL6ST** ]  ; i.e., a functional receptor complex of IL 6  [ **IL6** ]  type cytokines cannot be assembled solely via site I and II as in the growth hormone receptor complex.  Ref: 12731865 Biochemistry, 2003
- To define the IL 6  [ **IL6** ]  dependent intracellular pathways more specifically, we generated a hepatocyte specific gp130  [ **IL6ST** ]  knockout mouse.  Ref: 12891556 Gastroenterology, 2003
- Interleukin 6  [ **IL6** ]  IL 6  [ **IL6** ]  activates cells by binding to the membrane bound IL 6  [ **IL6** ]  receptor IL 6R and subsequent formation of a gp130  [ **IL6ST** ]  homodimer.  Ref: 12832423 J Biol Chem< MedlineTA>J Biol Chem, 2003
- TNF alpha Induces Tyrosine Phosphorylation and Recruitment of the Src Homology Protein Tyrosine Phosphatase 2 to the gp130  [ **IL6ST** ]  Signal Transducing Subunit of the IL 6  [ **IL6** ]  Receptor Complex.  Ref: 12817006 J Immunol, 2003
- Furthermore, activation of p38 mitogen activated protein kinase is shown to be essential for the inhibitory effect of TNF alpha on IL 6  [ **IL6** ]  signaling and TNF alpha dependent recruitment of SHP2 to gp130  [ **IL6ST** ] .  Ref: 12817006 J Immunol, 2003
- Thus, IL 6  [ **IL6** ]  gp130  [ **IL6ST** ]  signaling will become a more attractive therapeutic target for human inflammatory diseases when a better understanding of IL 6  [ **IL6** ]  signaling, including the identification of the conductor for gp130  [ **IL6ST** ]  signal transduction, is achieved.  Ref: 12687404 Rev Physiol Biochem Pharmacol< MedlineTA>Rev Physiol Biochem Pharmacol, 2003
- Epidermal growth factor receptor independent constitutive activation of STAT3 in head and neck squamous cell carcinoma is mediated by the autocrine paracrine stimulation of the interleukin 6  [ **IL6** ]  gp130  [ **IL6ST** ]  cytokine system.  Ref: 12782602 Cancer Res, 2003
- ELISA was used to determine the serum levels of soluble interleukin 2 receptors, interleukin 1, interleukin 6  [ **IL6** ] , and 2 soluble interleukin 6  [ **IL6** ]  receptors sIL 6R and soluble gp130  [ **IL6ST** ]  in 18 patients with cluster headache 6 women and 12 men during the cluster period and in 17 healthy controls who were headache free 3 women and 14 men.  Ref: 12864761 Headache, 2003
- Interleukin 6  [ **IL6** ]  gp130  [ **IL6ST** ]  dependent pathways are protective during chronic liver diseases.  Ref: 12830005 Hepatology< MedlineTA>Hepatology, 2003
- Since recruitment of the tyrosine phosphatase Src homology protein tyrosine phosphatase 2 SHP2 to the signal transducing receptor subunit gp130  [ **IL6ST** ]  attenuates IL 6  [ **IL6** ]  mediated STAT activation, we were interested in whether TNF alpha also induces the association of SHP2 to the gp130  [ **IL6ST** ]  receptor subunit.  Ref: 12817006 J Immunol, 2003
- Using the recently engineered designer cytokine IC7, which induces an active heterodimer of the LIFR and gp130  [ **IL6ST** ]  after binding to the IL 6R, and several receptor chimeras of the LIFR and the interleukin 6  [ **IL6** ]  receptor IL 6R carrying the CBM of the IL 6R in place of the COOH terminal LIFR CBM, we could assign individual receptor subdomains to individual binding sites of the ligand.  Ref: 12731865 Biochemistry, 2003
- Fibroblasts Isolated from Normal Lungs and Those with Idiopathic Pulmonary Fibrosis Differ in Interleukin 6  [ **IL6** ]  gp130  [ **IL6ST** ]  Mediated Cell Signaling and Proliferation.  Ref: 12819039 Am J Pathol, 2003
- In this context the cytoplasmic SHP2 SOCS3 recruitment site of gp130  [ **IL6ST** ]  tyrosine 759 is shown to be important for the inhibitory effects of TNF alpha, since mutation of this residue completely restores IL 6  [ **IL6** ]  stimulated activation of STAT3 and, consequently, of a STAT3 dependent promoter.  Ref: 12817006 J Immunol, 2003
- To prove the relevance of these clinical findings, we applied the tetrachlorcarbonide CCl 4 model to conditional knockout animals Cre loxP system for gp130  [ **IL6ST** ] , the common signal transducer of IL 6  [ **IL6** ]  family cytokines.  Ref: 12830005 Hepatology< MedlineTA>Hepatology, 2003
- In macrophages lacking the Socs3 gene or carrying a mutation of the SOCS3 binding site in gp130  [ **IL6ST** ] , the lipopolysaccharide induced production of tumor necrosis factor TNF and IL 12 is suppressed by both IL 10 and IL 6  [ **IL6** ] .  Ref: 12754507 Nat Immunol< MedlineTA>Nat Immunol, 2003
- Cells that express gp130  [ **IL6ST** ] , but not the IL 6R, can be activated by IL 6  [ **IL6** ]  and the soluble IL 6R sIL 6R which is generated by shedding from the cell surface or by alternative splicing.  Ref: 12832423 J Biol Chem< MedlineTA>J Biol Chem, 2003
- Although IL 6  [ **IL6** ]  gp130  [ **IL6ST** ]  signaling is a promising target for drug discovery for many human diseases, the interdependence of each signaling pathway may be an obstacle to the development of a nonpeptide orally active small molecule to inhibit one of these IL 6  [ **IL6** ]  signaling cascades, because it would disturb the signal orchestration.  Ref: 12687404 Rev Physiol Biochem Pharmacol< MedlineTA>Rev Physiol Biochem Pharmacol, 2003
- BACKGROUNDLeukemia inhibitor factor LIF, a member of the interleukin 6  [ **IL6** ]  cytokine family, has recently been shown to inhibit HIV 1 replication both in vivo and in vitro.OBJECTIVELIF and its corresponding receptors gp130  [ **IL6ST** ]  and LIF receptor alpha LIFR alpha were studied in lymphoid tissue LT to reveal potential systemic immunoregulatory effects during the course of HIV 1 infection.METHODSLIF, gp130  [ **IL6ST** ] , LIFR alpha and HIV 1 replicating cells were identified at the single cell level by immunohistochemistry and quantified by computerized in situ imaging in tonsil and lymph nodes biopsies LT from patients with primary HIV 1 infection PHI, chronic HIV 1 infection cHI, long term non progressors LTNP and HIV 1 seronegative controls.RESULTSLIF and its receptors, gp130  [ **IL6ST** ]  and LIFR alpha were significantly P &lt.  Ref: 12799551 AIDS< MedlineTA>AIDS, 2003
- Hexameric structure and assembly of the interleukin 6  [ **IL6** ]  IL 6  [ **IL6** ]  alpha receptor gp130  [ **IL6ST** ]  complex.  Ref: 12829785 Science, 2003
- after IL 6  [ **IL6** ]  and OSM stimulation, gp130  [ **IL6ST** ]  dependent pathways STAT3, APR gene expression were completely blocked in the liver of these animals.  Ref: 12891556 Gastroenterology, 2003
